# Genome sequences of two highly colistin-resistant *Pseudomonas aeruginosa* clinical isolates from the cystic fibrosis lung

**DOI:** 10.1128/mra.00558-25

**Published:** 2025-11-04

**Authors:** Anne E. Mattingly, Yohei Doi, Christian Melander

**Affiliations:** 1 Department of Chemistry and Biochemistry, University of Notre Dame 6111, Notre Dame, Indiana, USA; 2 Division of Infectious Diseases, Center for Innovative Antimicrobial Therapy, School of Medicine, University of Pittsburgh 6614, Pittsburgh, Pennsylvania, USA; University of Maryland School of Medicine, Baltimore, Maryland, USA

**Keywords:** colistin, *Pseudomonas aeruginosa*, antibiotic resistance, cystic fibrosis

## Abstract

The genomes of two highly colistin-resistant strains of *Pseudomonas aeruginosa* (MICs ≥ 1,024 mg/mL) isolated from cystic fibrosis lung infections were sequenced and analyzed. Genes associated with colistin resistance (*pmrAB*, *phoPQ*) showed unique mutations compared to other strains with similar resistance levels.

## ANNOUNCEMENT

Cystic fibrosis (CF) is a genetic disease marked by mucus build-up in airways with poor mucociliary clearance, creating an environment amenable to polymicrobial infections (1–3), from which *Pseudomonas aeruginosa* is frequently isolated (4). These *P. aeruginosa* infections are often treated with alternating courses of inhaled tobramycin or colistin (3, 5). We sequenced the genomes of two highly colistin-resistant strains of *P. aeruginosa* isolated from a bronchial wash specimen of a CF patient at the University of Pittsburgh Medical Center, collected under PRO12060302 as an exempt protocol approved by the University of Pittsburgh IRB, for use in a co-culture model to identify compounds with simultaneous antibiotic and adjuvant activities (6). Isolates were grown on TSA agar at 37°C for 24–48 h and identified by MicroScan Walkaway. Minimum inhibitory concentrations (MICs) for colistin were determined following CLSI standards for broth microdilution in Cation-adjusted Mueller-Hinton Broth (CAMHB, BD) (6, 7) (Table 1).

**TABLE 1:** Draft genome assembly details and statistics of *P. aeruginosa* clinical isolates from the same bronchial wash specimen^*a*^

Strain	TRPA161	TRPA162
Species	*P. aeruginosa*	*P. aeruginosa*
Colistin MIC (µg/mL)	8,192	1,024
Genome size (bp)	7,050,894	6,958,250
Total read coverage	400×	400×
Effective read length	2 × 150	2 × 150
No. of reads	3,894,656	3,710,459
% Bases ≥30	91.41	90.28
# of contigs	63	51
GC content (%)	60.76	61.66
N50 value (bp)	171,702	205,044
Mutations in PmrA	None	None
Mutations in PmrB	Y 345 H	Y 345 H
Mutations in PhoP	None	None
Mutations in PhoQ	DGELSLDDSELG 426-437 EGS**P**OKTENKAR E428_L429insP G438*	DGELSLDDSELG 426-437 EGS**P**OKTENKAR E428_L429insP G438*
Genome accession no.	JBLYTN000000000	JBLYTM000000000

^*a*^ “*” indicates stop codon and protein termination.

For whole-genome sequencing, strains were grown in CAMHB at 37°C for 18 h, pelleted, and stored at −80°C. DNA extraction and genome sequencing were conducted by Azenta Genewiz. DNA was extracted using the PureLink Genomic DNA Kit, fragmented by acoustic shearing, and prepared for libraries using NEBNext Ultra DNA Library Prep Kit for Illumina, following manufacturer recommendations. Libraries were sequenced on an Illumina MiSeq platform with a 2 × 150 paired-end sequencing configuration. Default parameters were used for all software unless otherwise specified. The paired-end samples were quality assessed via FastQC v0.11.5 (8). Raw data were quality trimmed using Trimmomatic v0.36 (9) with palindrome mode enabled, then down-sampled to 20 million reads. SPAdes v3.10 (10) *de novo* genome assembler was used on each sample. The assembled contigs were filtered for 500 bp. Quast v4.2 (11) was used to generate statistics for the assemblies. Genome annotation was completed using NCBI Prokaryotic Genome Annotation Pipeline v6.10 (12–14).

Colistin-resistance-associated genes *pmrAB* and *phoPQ* were analyzed using Geneious Prime 2025.0.2. These two-component systems are associated with the modification of lipid A, leading to colistin resistance in Gram-negative pathogens (15–20). Comparison to the *P. aeruginosa* PAO1 reference genome (NC_002516) showed significant mutations and predicted amino acid changes (Table 1). Notably, the last approximately 70 nucleotides of *phoQ* shared under 50% similarity to PAO1, predicting a truncated PhoQ protein. Mutations did not match other *P. aeruginosa* strains with similarly high colistin resistance (6, 20, 21), prompting further research into the mechanism by which these mutations contribute to colistin resistance.

## Data Availability

Whole-genome shotgun sequencing project and SRA of TRPA161 (JBLYTN000000000) and TRPA162 (JBLYTM000000000) are deposited in NCBI GenBank as Bioproject PRJNA1194588. The first versions are described in this paper. Sequences for genes specific to colistin-resistance mechanisms (pmrAB, phoPQ) are deposited in NCBI GenBank as PQ720584, PQ720585, PQ720586, and PQ720587 (TRPA161) and PQ720590, PQ720591, PQ720592, and PQ720593 (TRPA162).
